# Neglected tropical diseases in Pakistan: challenges, efforts, and recommendations

**DOI:** 10.1097/JS9.0000000000000186

**Published:** 2023-01-12

**Authors:** Humna Aamar, Javeria Arif Siddiqui, Amna Siddiqui, Mohammad Yasir Essar

**Affiliations:** a Faculty of Medicine, Sindh Medical College, Jinnah Sindh Medical University; b Department of Medicine, Karachi Medical and Dental College, Karachi, Pakistan; c Kabul University of Medical Sciences, Kabul, Afghanistan

*Dear Editor*,

In the early 2000s, neglected tropical diseases (NTDs) was coined as it refers to a set of diseases that occur in tropical countries due to the distinct geographical landscape, and distribution of the disease differs based on poverty in the region. Thus, they flourish in rural areas, conflict zones, and hard-to-reach regions, where there is generally a lack of quality healthcare, access to clean water, overcrowding, and scarce sanitation that is further aggravated by fluctuating climatic conditions. Pakistan ranks in the top 10 for the highest number of NTD cases for eight out of 18 NTDs researched in a study on global burden of disease^1^. Pakistan contributes greatly to the global disease burden of leishmaniasis, leprosy, trachoma, and soil-transmitted helminths^2^. Furthermore, other NTDs like dengue, chikungunya, and rabies are widespread in Pakistan, with case numbers increasing rapidly^3^,^4^. Though efforts have been made to eradicate these diseases, there have been no significant changes in the past 25 years^1^. Hence, this letter aims to discuss the challenges faced by the Pakistani population due to NTDs, the efforts made to eradicate these diseases, and recommendations to effectively control the prevalence of NTDs in the state.

The country faces an impending debt crisis and perennial trade imbalance on the economic front, combined with multiple internal and external conflicts rampant, causing 46% of the Pakistani population to fall below the poverty line^5^. Consequently, NTDs proliferate, further exacerbated by the lack of adequate treatment and prevention available for low- and middle-income families^1^. These diseases remain prevalent due to a plethora of reasons, such as due to poor sanitation conditions, overcrowding, and inaccessibility to healthcare. The climate of Pakistan, namely the arid desert and monsoon rains, provide the perfect environment for the disease agents to thrive^6^. Physical effects of NTDs include blindness, lesions, swelling, etc., which can disable a person from being able to see, walk, and live life normally^1^. Furthermore, amid the COVID-19 pandemic, NTD diagnosis and treatment is an additional burden on healthcare workers and organizations. As a result, there was a remarkable deviation in financial resources and reallocation of NTD personnel. Primarily, the pandemic has disrupted community-based interventions, that is, sanitation, health, and hygiene awareness campaigns. In addition, the pandemic has also hindered the production and deliverance of NTD medications to prevalent countries and in their distribution within governorates. Further, it has discontinued monitoring and evaluation activities like, that is, NTD surveillance and related surveys. Therefore, inadequate collection, analysis, and utilization of epidemiological data for planning purposes has further interrupted in successfully eradicating the NTDs, co-existent with the pandemic COVID-19 in the endemic state^7^.

In Pakistan, some efforts have been taken to control the spread of NTDs such as the International Trachoma Initiative (ITI). This initiative began in 2016 and in partnership with the Pakistan Ministry of Health, has started mass drug administration of azithromycin to treat trachoma^1^,^8^. This program has already shipped over one million doses of azithromycin in the years of 2016 and 2017, though was hindered by the pandemic in the past few years^8^. In 2017, the “Surgery, Antibiotics, Facial cleanliness, Environmental improvement SAFE strategy” was introduced in many regions of Pakistan through ITI^1^. The Federal Ministry of Health also attempted to expand its vector control activities against dengue, with some struggle due to weather events like seasonal flooding^1^. In addition, widespread multidrug therapy for leprosy given through existing treatment networks has begun with programs like Aid to Leprosy Patients (ALP) based in Rawalpindi, and Maria Adelaide Leprosy Centre (MALC) based in Karachi^1^. In 2019, Pakistan suffered from a dengue outbreak, with 47 120 confirmed cases of dengue and 75 deaths reported by the health authorities^2^. WHO helped combat this disease by spreading social awareness, management of disease, and vector-borne control of dengue^2^. Even with these efforts, the prevalence rates of Pakistan’s most common NTDs (e.g. soil-transmitted helminths, caseous lymphadenitis, trachoma, and leprosy) do not appear to have undergone significant changes over the last 25 years^1^. NTDs present a rising concern and a resolution made in 2013 by the World Health Assembly called for the integration of NTD diagnostic and treatment services into primary healthcare. The UN also stood to eradicate these diseases by 2030, by including the control, elimination, and eradication of NTDs in the Sustainable Development Goals (SDGs) introduced in 2015^9^. They are a group of global goals aimed at resolving and managing challenges to a “sustainable future for all”^2^. The SDGs also seek to end health inequities and increase access to healthcare, end poverty in all its forms, and ensure access to clean water and sanitation^4^. Action across any of these goals will facilitate the control and elimination of NTDs. Pakistan has committed to SDGs to combat disease and contribute to global health and development.

Eradication of these diseases can be achieved with an increase in monitoring, evaluation, and surveillance with proper diagnostic tools and actions to reduce transmission. Greater availability of health resources in Pakistan’s rural areas could reduce the health burden of these diseases greatly^2^. A global effort is required to ensure all people have access to clean, running water and educating the population on proper sanitation methods. The Government of Pakistan and its Federal Ministry of Health have implemented actions against vaccine-preventable childhood diseases, like Polio, with great success through the Expanded Programme on Immunisation (EPI)^10^. Therefore, integrating NTD treatment methods into such programmes could bring similar successes and bring Pakistan closer to eradicating NTDs. Though, for such a program to work, vaccine availability would need to be increased and be sufficiently distributed throughout Pakistan, especially to endemic regions. Moreover, improved vaccines for diseases like rabies, typhoid fever, and dengue, and biotechnologies to help in the diagnosis of disease are critical for the success of the program. Implementation of better sanitary practices are imperative as the majority of NTDs in Pakistan are spread through the fecal–oral route. Educating the public about good hand hygiene and NTDs would help decrease transmission and change the mindset of those who prefer home remedies over proper treatment. To combat against vector-borne diseases like dengue, awareness for prevention techniques like wearing long-sleeved clothing and using mosquito repellent are necessary. Through such efforts, and global help, the burden of NTDs in Pakistan can be greatly reduced.

## Ethics approval and consent to participate

None.

## Sources of funding

None.

## Authors’ contribution

M.Y.E. conceived the concept of the paper. H.A., J.A.S., and A.S. wrote the first draft of the manuscript. H.A. and M.Y.E. edited the second draft. All authors read and approved the final draft.

## Conflicts of interest disclosure

The authors declare that they have no financial conflict of interest with regard to the content of this report.

## Research registration unique identifying number (UIN)

None.

## Guarantor

Mohammad Yasir Essar.

## References

[1] Blum AJ Majid MF Hotez PJ . Pakistan: a nation held back by NTDs. PLoS Negl Trop Dis 2018;12:e0006751. 30335756 10.1371/journal.pntd.0006751 PMC6193611

[2] Khan A Tariq M Simsek S . Neglected tropical diseases in Pakistan: a story of neglect. Iran J Parasitol 2020;15:618–620. 33884021 10.18502/ijpa.v15i4.4882 PMC8039485

[3] Rauf M Fatima-Tuz-Zahra Manzoor S . Outbreak of chikungunya in Pakistan. Lancet Infect Dis 2017;17:258. 10.1016/S1473-3099(17)30074-9 28244384

[4] DAWN.COM. Anti-rabies project launched in Karachi – Newspaper. Accessed 20 November 2022. https://www.dawn.com/news/1382541

[5] Khaliq A . Pakistan in a perfect debt spiral with the worst impacts of the pandemic – CADTM. Accessed 20 November 2022. https://www.cadtm.org ; https://www.cadtm.org/Pakistan-in-a-perfect-debt-spiral-with-the-worst-impacts-of-the-pandemic

[6] Baqir M Sobani ZA Bhamani A . Infectious diseases in the aftermath of monsoon flooding in Pakistan. Asian Pac J Trop Biomed 2012;2 76. 10.1016/S2221-1691(11)60194-9 PMC3609207 23569839

[7] World Health Organization. Second round of the national pulse survey on continuity of essential health services during the COVID-19 pandemic: January-March 2021: interim report, 22 April 2021. 2021. Accessed 20 November 2022. https://apps.who.int/iris/handle/10665/340937

[8] International Trachoma Initiative. Pakistan. Accessed 20 November 2022. https://www.trachoma.org/where-we-work/pakistan

[9] Malecela MN . Reflections on the decade of the neglected tropical diseases. Int Health 2019;11:338–340. 31529110 10.1093/inthealth/ihz048

[10] Federal Directorate of Immunization, Pakistan. Polio. Accessed 20 November 2022. http://www.epi.gov.pk/vaccine-preventable-diseases/polio/

